# Effectiveness of Shoe Rotation in Managing Plantar Fasciitis in Patients

**DOI:** 10.3390/jcm13164624

**Published:** 2024-08-07

**Authors:** See-Won Koo, Yong-Soon Yoon, Myeong-Kwon Yoon, Seung-Gue Choi, Dong-Wuk Kim, Hong-Young Jang

**Affiliations:** 1 Department of Rehabilitation Medicine, Presbyterian (Jesus) Medical Center, Jeonju 54987, Republic of Korea; 9c19c1@hanmail.net (S.-W.K.); comayoon1@jesushospital.com (M.-K.Y.); mugapa3@jesushospital.com (S.-G.C.); dwkum09@jesushospital.com (D.-W.K.); 2 Innovation Division, Mokwon University, Daejeon 35349, Republic of Korea; brighthong0@mokwon.ac.kr

**Keywords:** plantar fasciitis, heel pain, footwear, treatment

## Abstract

**Background/Objectives:** Plantar fasciitis (PF) is a common condition that causes heel pain. While various conservative treatment modalities for PF exist, no previous studies have investigated the effectiveness of shoe rotation (ShR) in patients with PF pain. This study aimed to compare the therapeutic effectiveness of ShR with that of two conventional treatments for PF—namely, foot orthosis (FO) and physical therapy (PT). **Methods**: Charts of 42 patients with heel pain were retrospectively reviewed. Participants were allocated to one of three treatment groups: the ShR group, the customized FO group, and the PT group. Pain and functional outcomes were assessed using the Visual Analog Scale (VAS), Digital Pain Scale (DPS), Foot Function Index (FFI), Foot Pain and Function Scale (FPFS), and American Orthopedic Foot and Ankle Society Ankle-Hindfoot Scale (AOFAS-AHS) at baseline and at 4 and 12 weeks after the intervention. **Results**: The ShR, FO, and PT groups all showed improvements, with statistically significant decreases in VAS, DPS, and FFI scores and significant increases in FPFS and AOFAS-AHS scores over time (*p* < 0.05). All three interventions resulted in significant improvements from baseline to 4 weeks and further to 12 weeks (*p* < 0.05). The ShR group exhibited a slightly larger effect on all measurements than the other groups. **Conclusions**: ShR, FO, and PT contributed to pain reduction and functional improvement, and alternating the shoes alleviated PF pain. These results suggest a new approach to managing PF and serve as a basis for providing convenient treatment for patients with PF.

## 1. Introduction

Depending on the location of discomfort, heel pain syndrome is generally divided into plantar heel pain and posterior heel pain. Plantar fasciitis (PF) is a prevalent condition and is regarded as the most common cause of plantar heel pain in adults. In the United States, over a million people receive treatment for PF annually [1]. In South Korea, the prevalence rate of PF is approximately 10% across sexes [2].

The etiology of PF remains unclear, and multiple factors may play a role [3,4]. Excessive pronation has been recognized as one of the most common factors [4,5]. Other contributing factors include obesity, pes cavus foot, flat feet, limited ankle dorsiflexion due to shortened calf muscles or Achilles tendons, and inappropriate footwear [6,7].

Nonsurgical treatments for alleviating the symptoms of PF include physical therapy (PT) and foot orthosis (FO), which have been reported to be effective [8,9]. PT can mitigate pain and improve function through various methods such as pain modalities, therapeutic ultrasound, laser therapy, and calf muscle/plantar fascia stretching [10,11,12]. Orthopedic shoes and FO can reduce the pressure on the plantar fascia, contributing to long-term symptom reduction, functional improvement, and enhanced quality of life [8,13,14]. The effectiveness of orthotic devices in distributing pressure varies, depending on their design characteristics and on whether they are prefabricated or customized [14]. Some previous studies have indicated the beneficial effects of local steroid injection therapy and extracorporeal shock-wave therapy (ESWT) [6,15,16]. However, other studies have reported no significant effects when compared with sham treatments, resulting in divided opinions regarding their therapeutic efficacy [17].

Kim et al. analyzed data from the National Health Insurance Service of South Korea from 2007 to 2011 and reported seasonal variations in the incidence of PF. They observed that thicker and more cushioned footwear was commonly worn in winter and that the transition to shoes with flat heels and harder soles in summer led to an increase in the frequency of PF [4]. Furthermore, Rajput et al. reported the impact of routine footwear design on the onset of PF [18]. Nonetheless, the role of shoes in the treatment of PF is not well known [19,20].

A previous investigation regarding the influence of footwear on PF revealed that 83% of patients wore inappropriate shoes with low heels, thin soles, and hard insoles without any built-in arch support and that patients wearing improper footwear experienced severe heel pain [20]. Similarly, previous studies also demonstrated the significance of wearing correct shoes that provided adequate support and cushioning for patients with PF [4,19].

It is crucial to ensure that footwear fits correctly and that the heels and soles are not worn down in patients with PF [21]. Scher et al. (2010) [22] and Agyekum et al. (2015) [21] suggested that patients with PF should replace worn-out shoes to prevent exacerbating their condition. However, the effect of shoes without structural deformations on managing PF pain remains unverified.

In our clinical experience, patients with PF often complain about conventional treatments. They find FO expensive, are hesitant about frequent clinic visits for PT, and have concerns about invasive procedures. From this perspective, the concept of shoe rotation was designed as a cost-effective and convenient alternative to conventional treatments. This clinical intervention involves patients purchasing and alternately wearing new pairs of sneakers to replace their old ones. By eliminating the need for hospital visits, shoe rotation offers an easily accessible and affordable solution, and high management compliance is therefore expected among patients.

The present study aimed to determine whether alternating shoes daily could significantly alleviate pain in patients with PF and to investigate the effects of shoe rotation (ShR) among different pairs of shoes on the management of PF by comparing the clinical effectiveness of three treatment methods—namely, ShR, FO, and PT.

## 2. Materials and Methods

### 2.1. Participants and Criteria

A retrospective chart review was conducted on outpatients following the approval of the Institutional Review Board (IRB) of the Presbyterian Medical Center (approval number: 2023-11-047; approval date: 12 December 2023). The IRB waived the requirement for informed consent owing to the retrospective nature of the study.

Medical records of patients who visited the Department of Rehabilitation Medicine at the Presbyterian Medical Center from January 2010 to October 2015 were collected. The inclusion criteria were as follows: individuals aged > 40 years (i) who had unilateral plantar heel pain and tenderness in the infra-calcaneal region on physical examination; (ii) who expressed a desire for pain alleviation; (iii) who were capable of adhering to instructions; and (iv) who exhibited wear patterns on the inside edge of the heel and the inside of the sole in their shoes. The exclusion criteria were as follows: (i) fractures involving the calcaneus, talus, or other bones of the foot; (ii) comorbidities such as lumbar radiculopathy; (iii) suspicion of ankle ligament damage; (iv) a history of recent treatments for PF within 3 months and foot or ankle surgeries within 6 months; (v) severe cognitive or behavioral disorders; (vi) inability to detect temperature changes; (vii) malignant tumors or skin diseases; (viii) current use of nonsteroidal anti-inflammatory drugs (NSAIDs); and (ix) any other conditions deemed unsuitable by the principal investigator.

### 2.2. Interventions

A total of 42 participants were analyzed. Fourteen patients were allocated to the ShR group, customized FO group, and PT group, respectively, with each group receiving different treatments. None of the participants received any other treatments.

In the ShR group, the participants were asked to choose three new pairs of sneakers: regular cushioned sneakers that were not functional or running shoes. The choice of footwear was left to the patients. The participants were advised to select general sneakers that fitted properly and were comfortable, regardless of whether they were the same model as their previous sneakers. The participants were instructed to rotate through the three pairs of shoes, wearing one pair each day for three days and then repeating the cycle by wearing the first pair again on the fourth day. This therapeutic intervention was maintained for 12 weeks. The participants in the FO group applied customized insoles using the Triple-Pod System. Patients underwent initial assessment standing on a plantar pressure monitor, and based on the data obtained, insoles were custom molded to fit their feet. The participants in the PT group received pain modalities. The treatment involved infrared therapy for 15 min, transcutaneous electrical nerve stimulation (TENS) for 20 min, and microwave therapy for 30 min three times a week. All participants visited the clinic at baseline, 4 weeks, and 12 weeks during treatment. During each visit, relief from PF pain was compared between the three groups using the following clinical assessment tools.

### 2.3. Clinical Assessments

For pre-evaluation (visit 1), medical records were reviewed to obtain information on the participants’ age, sex, duration, and characteristics of heel pain (primary lesion side, intensity, etc.), body mass index (BMI), comorbidities, and history and duration of treatments. Eligibility screening had been conducted on participants who consented to the treatment.

For treatment evaluation (visits 2–4), all parameters were evaluated at baseline prior to the initiation of the clinical intervention and then at 4 and 12 weeks. The primary outcome of foot pain and function was evaluated using the Visual Analog Scale (VAS), Digital Pain Scale (DPS), Foot Function Index (FFI), Foot Pain and Function Scale (FPFS), and American Orthopedic Foot and Ankle Society Ankle-Hindfoot Scale (AOFAS-AHS). The DPS was assessed using a digital pressure algometer (Commander Echo Algometer; J-TECH Medical, Midvale, UT, USA). Vital signs, concomitant medications, and adverse reactions were monitored.

### 2.4. Statistical Analysis

Data analyses were performed using SPSS for Windows version 28.0 (IBM Corp., Armonk, NY, USA). Homogeneity tests for sociodemographic and general characteristics among the three groups (ShR, FO, and PT) were performed using the chi-squared test and one-way ANOVA. Additionally, differences in the mean values of pain and functional assessment tools (VAS, DPS, FFI, FPFS, and AOFAS-AHS) across treatment timelines (baseline, 4 weeks, and 12 weeks) were evaluated using repeated-measures ANOVA. Statistical significance was set at *p* < 0.05 for all tests.

Before interpreting the results, Mauchly’s test for sphericity was performed. If the assumption of sphericity was met, the corresponding values were utilized; if not, corrections for within-subject effects were applied based on Mauchly’s ε values. The Huynh–Feldt estimates were used for *W* values above 0.75, whereas Greenhouse–Geisser estimates were applied for *W* values below 0.75.

## 3. Results

### 3.1. General Characteristics

A total of 42 participants divided into three groups were evaluated. The ShR group had an average age of 56.07 ± 5.70 years, with six male and eight female patients. The FO group had an average age of 54.71 ± 5.04 years, with five male and nine female patients. The PT group had an average age of 55.36 ± 4.31 years, with four male and ten female patients. No differences in sex, primary lesion side, Achilles scale score, age, duration of symptoms, and BMI_pre or BMI_post were observed, confirming that the three groups were comparable (*p* > 0.05) (Table 1).

**Figure 1:**
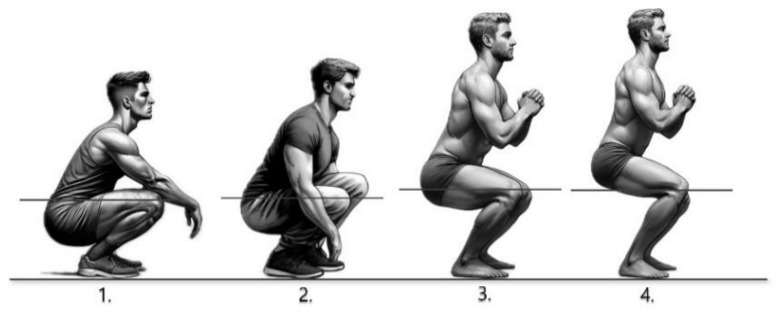
Preparation postures for the evaluation of our proposed Achilles scale. From right to left, scores from 4 to 1 are assigned based on the point at which the heel rises off the ground as the participant descends into a deeper squat. A higher score indicates higher severity of PF. Participant 1 is depicted in a fully crouched position, whereas participant 3 assumes a natural squatting stance. The line represents the level of the knees.

The Achilles scale was designed to assess the severity of PF. On this scale, scores ranging from 1 to 4 were assigned based on the point at which the heel lifted off the ground during a squat (Figure 1).

### 3.2. VAS and DPS Scores

**Figure 2:**
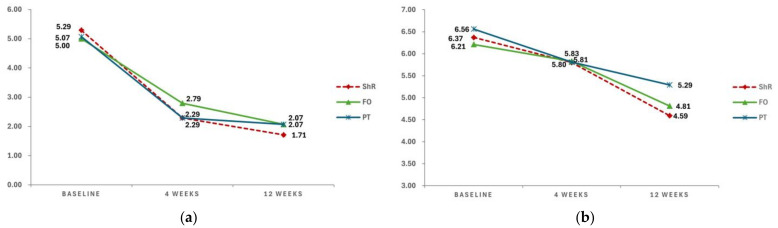
(**a**) Changes in the mean VAS scores. (**b**) Changes in the mean DPS scores. (VAS, Visual Analog Scale; DPS, Digital Pain Scale; ShR, shoe rotation; FO, foot orthosis; PT, physical therapy).

The VAS and DPS scores were assessed in the ShR, FO, and PT groups at baseline, 4 weeks, and 12 weeks (Figure 2).

The VAS scores showed gradual improvement with treatment in all three groups. In the ShR group, the VAS scores improved at 4 weeks (2.29 ± 0.61) and 12 weeks (1.71 ± 0.99) compared with baseline (5.29 ± 0.83). In the FO group, the VAS scores improved at 4 weeks (2.79 ± 0.58) and 12 weeks (2.07 ± 1.21) compared with baseline (5.00 ± 0.68). Similarly, in the PT group, the VAS scores improved at 4 weeks (2.29 ± 0.61) and 12 weeks (2.07 ± 1.00) compared with baseline (5.07 ± 0.83).

The DPS scores also gradually improved with treatment in all three groups. In the ShR group, the DPS scores improved at 4 weeks (5.80 ± 0.97) and 12 weeks (4.59 ± 0.94) compared with baseline (6.37 ± 0.60). In the FO group, the DPS scores improved at 4 weeks (5.83 ± 0.72) and 12 weeks (4.81 ± 1.22) compared with baseline (6.21 ± 0.98). Similarly, in the PT group, the DPS scores improved at 4 weeks (5.81 ± 0.93) and 12 weeks (5.29 ± 0.89) compared with baseline (6.56 ± 0.71).

To determine statistical significance, a repeated-measures ANOVA was performed using the mean VAS and DPS scores as dependent variables (Table 2).

Significant differences in the VAS and DPS scores were observed over time (*p* < 0.001); however, no statistically significant interaction effects were observed between time and group. Furthermore, no significant differences were noted among the groups. Overall, the VAS and DPS scores significantly improved over time across the ShR, FO, and PT groups. Notably, the effects observed in the ShR group were relatively greater than those observed in the other groups.

### 3.3. FFI and FPFS Scores

**Figure 3:**
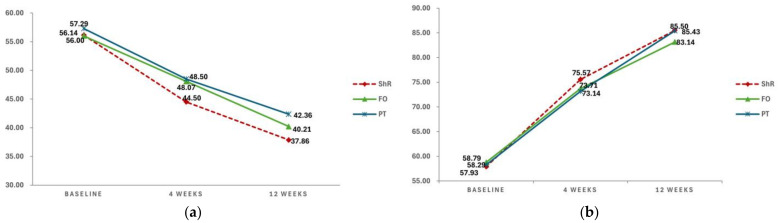
(**a**) Changes in the mean FFI scores. (**b**) Changes in the mean FPFS scores. (FFI, Foot Function Index; FPFS, Foot Pain and Function Scale; ShR, shoe rotation; FO, foot orthosis; PT, physical therapy).

The FFI and FPFS scores were assessed in the ShR, FO, and PT groups at baseline, 4 weeks, and 12 weeks (Figure 3).

The FFI scores exhibited gradual improvement with treatment in all three groups. In the ShR group, the FFI scores improved at 4 weeks (44.50 ± 6.93) and 12 weeks (37.86 ± 4.15) compared with baseline (56.14 ± 2.93). In the FO group, the FFI scores improved at 4 weeks (48.07 ± 5.00) and 12 weeks (40.21 ± 4.35) compared with baseline (56.00 ± 3.66). Similarly, in the PT group, the FFI scores improved at 4 weeks (48.50 ± 6.44) and 12 weeks (42.36 ± 3.13) compared with baseline (57.29 ± 3.22).

A gradual improvement in the FPFS scores was observed with treatment in all three groups. In the ShR group, the FPFS scores improved at 4 weeks (75.57 ± 2.14) and 12 weeks (85.50 ± 3.46) compared with baseline (57.93 ± 4.23). In the FO group, the FPFS scores improved at 4 weeks (73.71 ± 1.59) and 12 weeks (83.14 ± 1.79) compared with baseline (58.79 ± 4.73). Similarly, in the PT group, the FPFS scores improved at 4 weeks (73.14 ± 2.07) and 12 weeks (85.43 ± 2.82) compared with baseline (58.29 ± 3.56).

To determine statistical significance, a repeated-measures ANOVA was performed using the mean FFI and FPFS scores as dependent variables (Table 3).

Significant differences in the FFI and FPFS scores were observed over time (*p* < 0.001); however, no statistically significant interaction effects were detected between time and group. There was a significant difference in FFI scores across the groups (*p* < 0.05); in contrast, no significant difference in FPFS scores was noted across the groups. Overall, the FFI and FPFS scores significantly improved over time across the ShR, FO, and PT groups, but the interaction effects were not significant. Notably, the effects observed in the ShR group were relatively greater than those observed in the other groups.

### 3.4. AOFAS-AHS Scores

**Figure 4:**
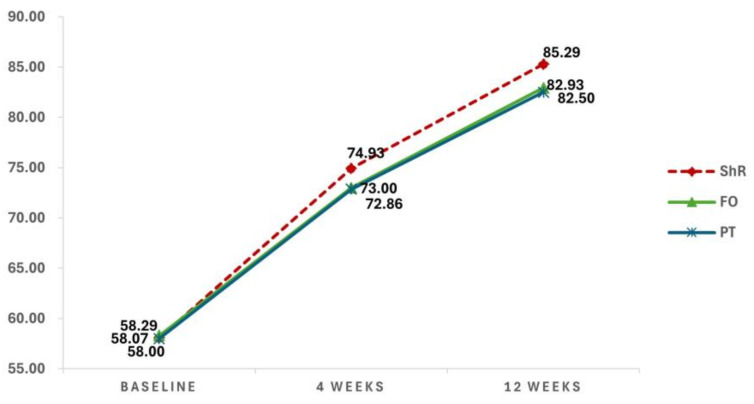
Changes in the mean AOFAS-AHS scores. (AOFAS-AHS, American Orthopedic Foot and Ankle Society Ankle-Hindfoot Scale; ShR, shoe rotation; FO, foot orthosis; PT, physical therapy.)

The AOFAS-AHS scores were assessed in the ShR, FO, and PT groups at baseline, 4 weeks, and 12 weeks (Figure 4).

The AOFAS-AHS scores gradually improved with treatment in all three groups. In the ShR group, the AOFAS-AHS scores improved at 4 weeks (74.93 ± 2.09) and 12 weeks (85.29 ± 2.84) compared with baseline (58.07 ± 3.05). In the FO group, the AOFAS-AHS scores improved at 4 weeks (73.00 ± 1.88) and 12 weeks (82.93 ± 2.06) compared with baseline (58.29 ± 3.85). Similarly, in the PT group, the AOFAS-AHS scores improved at 4 weeks (72.86 ± 1.88) and 12 weeks (82.50 ± 2.03) compared with baseline (58.00 ± 2.63).

To determine statistical significance, a repeated-measures ANOVA was performed using the mean AOFAS-AHS scores as dependent variables (Table 4).

Significant differences in the AOFAS-AHS scores were observed over time (*p* < 0.001); however, no statistically significant interaction effect was detected between time and group. A significant difference across the groups was found (*p* < 0.01). Overall, the AOFAS-AHS scores significantly improved over time across the ShR, FO, and PT groups, but the interaction effect was not significant. Notably, the effect observed in the ShR group was relatively greater than that observed in the other groups.

## 4. Discussion

In 1954, Hicks proposed the windlass mechanism as the source of biomechanical stress on the plantar fascia [2,23]. Excessive tensile forces repeatedly applied to the attachment of the plantar fascia are believed to trigger inflammation and degenerative changes [24,25]. An inappropriate foot arch (i.e., either a higher- or lower-arched foot) and excessive pronation are closely linked to the severity of PF, specifically related to the degeneration of the collagen fibers of the plantar fascia and its chronic hypertrophy [5,26,27]. Furthermore, inappropriate footwear that lacks foot mobility or causes excessive pronation, such as small and rigid shoes, has been identified as a biomechanical factor contributing to PF [2]. A 2014 heel pain survey conducted by the American Podiatric Medical Association indicated that wearing ill-fitting or uncomfortable shoes caused heel pain in 45% of respondents [28]. Additionally, elevating the heel height to a certain level (>4 cm) reportedly reduces the pressure on the heel and plantar fascia, thereby relieving heel pain [29].

Recent studies have identified excessive pronation as a factor closely related to the onset of PF [7,19]. This principle is explained by a decrease in the medial longitudinal arch that induces greater foot mobility, which promotes greater rearfoot pronation to maintain the stability of the subtalar joint. This results in a greater overload on the medial region of the calcaneus, producing greater stress on the plantar fascia and consequently contributing to the progression of PF [19]. Inappropriate shoes can exacerbate pronation [4], thereby increasing the stress on the foot [19,30] and ultimately generating PF [4,19]. However, research remains scarce regarding the association between shoes and PF, particularly on how deformations in the insoles of well-worn shoes can lead to structural changes and degenerative foot deformities, thereby influencing biomechanical alterations in the plantar fascia.

Agyekum et al. (2015) [21] reported that shoes can help prevent degenerative PF of the heel by absorbing some of the stress applied to the heel. The sole and heel of old shoes start to wear out with continual use [21]. This deterioration reduces the shoes’ capacity to absorb and disperse ground reaction forces. Consequently, most of the ground reaction forces are transmitted directly to the plantar fascia rather than the shoes, placing more stress and compressive forces on the feet and plantar aponeurosis [21,31].

Among the biomechanics of the PF, continuous and repetitive impacts on the inferior aspect of the calcaneus lead to an accumulated load on the bone, which progressively weakens the heel pad. This weakening allows for persistent micro-irritation at the site where the medial band of the plantar fascia attaches to the medial calcaneal tubercle, causing degeneration of the collagen fibers and leading to inflammation and pain in the plantar fascia [32]. Therefore, changes in shoes due to wear lead to microtears in the plantar fascia due to overloading [22].

Factors influencing the impact of plantar aponeurosis include the type of movement, landing speed, shoe structure, friction surface, and body mass [33]. From the perspective of impact factors, if the sole of a shoe becomes hard owing to wear or if the structure of the insole deforms, it can increase the impact forces on the foot, potentially triggering PF [4]. Shoe soles and heels are also known to reduce the ability to absorb and dissipate ground-reaction forces, thereby transferring increased stress to the plantar fascia [19]. Therefore, the findings of our study suggest that rotating shoes may delay shoe deformation and reduce loading on the origin of the calcaneus, thereby lowering the risk of developing PF.

PF is prone to recurrence, making it essential to focus on treatments that address the underlying causes rather than merely aiming for temporary symptom relief. Conservative treatment lasting over six months can yield favorable outcomes in more than 90% of cases.

Previous studies have compared and analyzed the effectiveness of different treatments for PF [12,34,35]. Li et al. (2018) [36] investigated the effects of different PT methods and found that TENS, ultrasound, and laser therapies were effective in improving pain and function in PF [37]. Yelverton et al. (2019) [38] examined the effects of manual therapy within PT and reported that manual therapy significantly enhanced the flexibility of plantar fascia tissues and alleviated pain. Petrofsky et al. (2020) [11] studied the effects of local heat therapy on Achilles tendinitis and reported its effectiveness in relieving the pain associated with PF.

Several studies have demonstrated the therapeutic effects of FO [39,40]. Bishop et al. (2018) [41] found that the combined use of a customized FO with new shoes was more effective in reducing PF pain and decreasing plantar fascia thickness than using new shoes alone. Martinez-Rico et al. (2020) confirmed that the application of plantar orthotics minimizes the stress and pressure on the PF and that this pressure distribution effect varies depending on the shape of the orthotics [42]. Choo et al. (2020) [43] verified that specialized shoes and various FOs are effective in reducing pain in conditions associated with heel pain.

Steroid injections, which are commonly used to treat patients with PF, have limited use because of their adverse effects [44]. Furthermore, the superiority of ESWT and injection therapies over other treatments has not been proven.

This study intends to enhance the reliability of research by analyzing the effectiveness of managing PF through a multifaceted evaluation. A digital pressure algometer provides an objective assessment of pressure pain thresholds [45]. The FFI and FPFS were used to acquire more nuanced measurements of the characteristic pain and function associated with PF [46]. The validity and reliability of the FFI have been demonstrated in several studies [9,47]. The FPFS comprises five questions regarding pain and five questions regarding function. The AOFAS was developed to analyze anatomical regional assessments of the foot in terms of pain (40%), function (50%), and alignment (10%) [48]. The AOFAS-AHS demonstrated acceptable criterion validity and adequate reliability for its subjective aspects [49,50]. All three groups showed improvements in pain-related indicators of PF pain after 12 weeks of intervention, with ShR demonstrating the most significant improvement. In addition, the sex and age of participants have been shown not to be significantly related to the outcome of the study. The recent literature reports that the relationship between sex and PF is inconsistent [22]. Some of the literature indicates a higher prevalence in females [51], some shows an increased prevalence in males [52], and other studies suggest that the association between sex and PF is still unclear [53]. Age is regarded as one of the intrinsic factors of PF, with peak incidence seen in people aged 40 to 60 in the general population [54].

The primary objective of this study was to propose a simple yet effective treatment approach for PF and contribute to the standardization of pain alleviation therapies. We verified that alternating everyday footwear alone significantly reduced the discomfort and pain associated with PF.

Rotating shoes is technically straightforward and easy to implement and offers a universally applicable treatment option without complications for all patients with PF. Treatments involving FOs and specialized shoes, such as rocker shoes, can pose a financial burden and may initially feel unnatural. By contrast, ShR is cost-effective, allows for easy-to- wear shoes, and is highly accessible. Specialized shoes may restrict activities such as long-distance running owing to their specific style or size and may not align with personal fashion preferences. However, regular sneakers do not impose such limitations, allowing wearers to match any clothing style without constraints on their daily lives. Additionally, ShR presents significantly fewer risks of complications or side effects than conventional treatments such as ESWT or pain modalities.

The proper use of shoes can protect patients’ feet from degeneration caused by the structural deformation of footwear, improve patients’ quality of life, and contribute to the development of cost-effective treatment methods for PF. These findings could be used by medical institutions to prescribe shoe rotation for patients with PF, thereby providing convenient and personalized therapeutic options that serve as valuable information for managing PF.

This study serves as an initial step toward an evaluation of the role of ShR in alleviating PF pain. Therefore, we address its limitations and outline future research directions. We initially confirmed that the participants wore regular sneakers prior to study commencement; however, we did not account for individual variations in their shoes. Because controlling every patient’s regular sneakers in a clinical setting is impractical, it could reduce sampling bias, which can potentially occur when standardizing shoes. Future studies that consider various types of shoes may allow for the acquisition of qualitative data on the effects of ShR based on differences in shoe characteristics. Future research should strengthen the validity of the ShR treatment effect identified in this study by conducting a controlled study comparing the management of PF in a group wearing alternating shoes and a group wearing a single pair of shoes. Additionally, while this study relied on the subjective reporting of symptoms by patients, future research should consider using imaging techniques, such as ultrasound or radiography, as evaluation tools to objectively assess structural changes in the plantar fascia and foot.

Although we observed changes over a 12-week treatment period, further research is necessary to evaluate the long-term effects and recurrence rates of PF during ShR management. By gathering sufficient registration trial numbers of participants and investigating the effects of long-term treatment over six months or more, a deeper understanding of the pain relief efficacy of this approach can be achieved. Such studies could clarify the therapeutic value of ShR and enhance the qualitative improvement of PF management.

## 5. Conclusions

Shoe rotation, foot orthosis, and physical therapy contributed to pain reduction and functional improvements in patients with PF following the 12-week intervention. Notably, simply rotating the shoes significantly alleviated PF pain. Shoe rotation involves understanding the role of footwear in PF treatment, and is a cost-effective and non-invasive alternative to conventional interventions. These results suggest a new approach to managing PF and serve as a basis for providing convenient treatment options for patients with PF.

## Figures and Tables

**Table 1:** Homogeneity tests for general characteristics.

Variables		ShR (*n* = 14) *n* (%)	FO (*n* = 14) *n* (%)	PT (*n* = 14) *n* (%)	χ^2^	*p*
Sex	Male	6 (42.9)	5 (35.7)	4 (28.6)	0.622	0.733
	Female	8 (57.1)	9 (64.3)	10 (71.4)		
Primary lesion	Right	5 (35.7)	6 (42.9)	5 (35.7)	0.202	0.904
	Left	9 (64.3)	8 (57.1)	9 (64.3)		
Achilles scale score	1	2 (14.3)	2 (14.3)	2 (14.3)	3.100	0.796
	2	5 (35.7)	4 (28.6)	6 (42.9)		
	3	7 (50.0)	8 (57.1)	5 (35.7)		
	4	0 (0.0)	0 (0.0)	1 (7.1)		
**Demographics**		**ShR** **M ± SD**	**FO** **M ± SD**	**PT** **M ± SD**	***F***	***p***
Age (years)		56.07 ± 5.70	54.71 ± 5.04	55.36 ± 4.31	0.253	0.778
Symptom duration (months)		5.71 ± 0.73	5.64 ± 0.63	5.79 ± 0.80	0.136	0.873
BMI_pre		26.28 ± 2.83	27.34 ± 5.35	26.38 ± 2.74	0.328	0.722
BMI_post		26.12 ± 2.47	27.36 ± 5.30	26.48 ± 2.87	0.406	0.669

Values are presented as mean (SD). Data were analyzed using the chi-squared test and one-way ANOVA. (ShR, shoe rotation; FO, foot orthosis; PT, physical therapy.)

**Table 2:** Comparison of the VAS and DPS scores.

Variables	Source	Sum of Squares (SS)	Degrees of Freedom (df)	Mean Square (MS)	*F*	*p*
VAS	Time	243.444	2	121.722	192.825 ***	0.000
	Time * group	3.317	4	0.829	1.314	0.272
	Group	0.825	2	0.413	0.483	0.621
DPS	Time	47.200	1.866	25.296	48.986 ***	0.000
	Time * group	2.182	3.732	0.585	1.132	0.347
	Group	2.287	2	1.143	0.778	0.466

The asterisk (*) is used to denote the interaction effect between time and group. *** *p* < 0.001.

**Table 3:** Comparison of the FFI and FPFS scores.

Variables	Source	Sum of Squares (SS)	Degrees of Freedom (df)	Mean Square (MS)	*F*	*p*
FFI	Time	5648.619	2	2824.310	182.300 ***	0.000
	Time * group	70.952	4	17.738	1.145	0.342
	Group	219.857	2	109.929	3.305 **	0.047
	Duncan	ShR < PT				
FPFS	Time	14,782.492	1.517	9744.060	675.229 ***	0.000
	Time * group	73.698	3.034	24.290	1.683	0.180
	Group	26.968	2	13.484	1.858	0.169

The asterisk (*) is used to denote the interaction effect between time and group. ** *p* < 0.05, *** *p* < 0.001.

**Table 4:** Comparison of the AOFAS-AHS scores.

Variables	Source	Sum of Squares (SS)	Degrees of Freedom (df)	Mean Square (MS)	*F*	*p*
AOFAS-AHS	Time	13,816.048	1.585	8718.296	986.430 ***	0.000
	Time * group	36.381	3.169	11.479	1.299	0.283
	Group	64.714	2	32.357	5.750 **	0.006
	Duncan	PT, FO < ShR				

The asterisk (*) is used to denote the interaction effect between time and group. ** *p* < 0.01, *** *p* < 0.001.

## Data Availability

Data will be available upon reasonable request to the authors.
